# Succinylation modification provides new insights for the treatment of immunocompromised individuals with drug-resistant *Aspergillus fumigatus* infection

**DOI:** 10.3389/fimmu.2023.1161642

**Published:** 2023-04-17

**Authors:** Xianzhen Chen, Wenzhi Lei, Hui Meng, Yi Jiang, Sanli Zhang, Huyan Chen, Mingwei Du, Xiaochun Xue

**Affiliations:** ^1^ Institute of Dermatology, Naval Medical University, Shanghai, China; ^2^ Department of Dermatology, Central Hospital Affiliated to Shandong First Medical University, Jinan, China; ^3^ Department of Pharmacy, 905th Hospital of People's Liberation Army of China (PLA) Navy, Shanghai, China; ^4^ Department of Nephrology, Second Affiliated Hospital of Naval Medical University, Shanghai, China; ^5^ Department of Dermatology, Huashan Hospital, Fudan University, Shanghai, China; ^6^ Department of Cardiology, Shuguang Hospital Affiliated to Shanghai University of Traditional Chinese Medicine, Shanghai, China; ^7^ Shanghai Key Laboratory of Traditional Chinese Clinical Medicine, Shanghai, China

**Keywords:** immunocompromised, macrophage, drug resistance, *Aspergillus fumigatus*, succinylation

## Abstract

Invasive *Aspergillus fumigatus* infection poses a serious threat to global human health, especially to immunocompromised individuals. Currently, triazole drugs are the most commonly used antifungals for aspergillosis. However, owing to the emergence of drug-resistant strains, the effect of triazole drugs is greatly restricted, resulting in a mortality rate as high as 80%. Succinylation, a novel post-translational modification, is attracting increasing interest, although its biological function in triazole resistance remains unclear. In this study, we initiated the screening of lysine succinylation in *A. fumigatus*. We discovered that some of the succinylation sites differed significantly among strains with unequal itraconazole (ITR) resistance. Bioinformatics analysis showed that the succinylated proteins are involved in a broad range of cellular functions with diverse subcellular localizations, the most notable of which is cell metabolism. Further antifungal sensitivity tests confirmed the synergistic fungicidal effects of dessuccinylase inhibitor nicotinamide (NAM) on ITR-resistant *A. fumigatus*. *In vivo* experiments revealed that treatment with NAM alone or in combination with ITR significantly increased the survival of neutropenic mice infected with *A. fumigatus*. *In vitro* experiments showed that NAM enhanced the killing effect of THP-1 macrophages on *A. fumigatus* conidia. Our results suggest that lysine succinylation plays an indispensable role in ITR resistance of *A. fumigatus*. Dessuccinylase inhibitor NAM alone or in combination with ITR exerted good effects against *A. fumigatus* infection in terms of synergistic fungicidal effect and enhancing macrophage killing effect. These results provide mechanistic insights that will aid in the treatment of ITR-resistant fungal infections.

## 1 Introduction

Invasive aspergillosis (IA) has become an important cause of morbidity and mortality in immunocompromised patients (1) and is a major health concern for patients who may suffer hospital-related infections (2, 3). It is estimated that 250,000 cases of IA occur worldwide each year, with a mortality rate of 30%–80% (4). *Aspergillus fumigatus* is the most common pathogen for IA, accounting for approximately 80%–90% of cases. When the host’s immunity is compromised, such as through the frequent use of antibiotics, immunosuppressants, and chemotherapy drugs, *A. fumigatus* can invade the human body through the lungs or skin and cause fatal invasive infections (5–7).

Triazole antifungals that inhibit ergosterol biosynthesis and decrease Cyp51 lanosterol demethylase activity are the first-line therapies for invasive *A. fumigatus* infections (8). Compared with amphotericin B, triazole drugs are beneficial as they have higher clinical efficacy and fewer adverse reactions. The most commonly used triazole antifungals are itraconazole (ITR) and voriconazole (9). However, triazole-resistant strains of *A. fumigatus* are increasing at a concerning rate (10, 11), with 16.15% of *A. fumigatus* isolates showing resistance to at least one azole. This complicates treatment and increases fatality rates (12, 13). Most published studies concur that the original mutations most likely occurred because of the usage of azole-based fungicides in agriculture, as hypothesized by Snelders et al. However, the origin of these resistant alleles is yet unknown (14, 15).

Post-translational modification (PTM) of proteins is an efficient and rapid biological regulatory mechanism that links metabolism to protein and cell functions. PTMs include phosphorylation, acetylation, glycosylation, and succinylation (16–18); these can modulate a wide variety of cellular processes in eukaryotic and prokaryotic organisms, including cell growth and division, adaptation to environmental changes, and developmental guidance. Lysine succinylation, a frequently observed PTM, can be identified using high throughput high performance liquid chromatography–tandem mass spectrometry (LC–MS/MS) and antibody-based affinity enrichment. It has also been associated with significant structural alterations in proteins (19). With the rapid development of MS, lysine succinylation has been widely identified to exist in eukaryotes and prokaryotes, including *Saccharomyces cerevisiae, A. flavus*, and *Homo sapiens* (20–22). However, this complex PTM in *A. fumigatus* infection has been poorly studied.

Drug resistance of *A. fumigatus* can be caused by various mechanisms, including intrinsic aberrant gene expression and external environment effectors (23). Drug resistance is significantly influenced by acetylation and deacetylation (24), phosphorylation and dephosphorylation (25), succinylation and desuccinylation (26), and glycosylation and deglycosylation (27). Lysine succinylation is a newly identified protein PTM that is vital in cellular physiology. However, the role of lysine succinylation in fungal resistance remains unclear. Understanding the succinylation may assist clinical strategies and drug development for *A. fumigatus* infection.

As the initial step in understanding the function of lysine succinylation in ITR-resistant fungus, we used an integrated proteomic method in this study. Subsequently, *in vivo* and *in vitro* experiments were conducted to validate the effect of nicotinamide (NAM), a desuccinylation inhibitor, on fungal resistance and macrophage killing effect. Our findings have implications for antifungal therapy in immunocompromised patients.

## 2 Materials and methods

### 2.1 Strains and culture conditions

AF293, a sensitive *A. fumigatus* strain, was obtained from the American Type Culture Collection. The drug-resistant strains used in the present study were screened from the clinical strains kindly provided by the Changzheng Hospital in Shanghai. All strains were identified through internal transcribed spacer (ITS) DNA sequencing. The strains were retrieved from glycerol stocks frozen at -80°C and subcultured onto potato dextrose agar (PDA) plates and incubated at 35°C for 7 d before use. The conidial suspensions were dislodged from the hyphal mats through dispersal in phosphate buffered solution (PBS) with 0.05% Tween 80 and filtered through a 70-μm cell strainer. The strain was cultured at 28°C in the sabouraud dextrose broth medium at 200 rpm for 7 d to harvest the fungal hyphae. The hypha samples were stored in the −80°C freezer for the further experiments.

### 2.2 Microdilution assay

*In vitro* antifungal susceptibility testing was performed according to the M38-A2 protocol of the Clinical and Laboratory Standards Institute (CLSI) (28). Briefly, to each well of the 96-well microplates, 100 μL of the RPMI1640 medium containing gradiently-diluted antifungal agents were added. Subsequently, the prepared spore suspensions were added to drug-sensitive plates at concentrations of 1 × 10^5^ cells/well, and the microplates were incubated for 48 h at 35°C. The antifungal agents used in the study included ITR, voriconazole, and posaconazole, at a final concentration range of 0.03–16 μg/mL. All the antifungal reagents were purchased from Sigma-Aldrich (Shanghai) Trading Co, Ltd.

### 2.3 Protein extraction

The sample was taken out of the −80°C freezer and thawed on ice, then centrifuged at 5,500 ×*g* for 10 min. The supernatant was transferred into a new tube, and the precipitate was transferred to a mortar precooled by liquid nitrogen. Each group of samples was added to the supernatant solution and sonically lysed. An equal volume of Tris equilibrium phenol was added and centrifuged at 5,500 ×*g* for 10 min at 4°C, then take the supernatant and add 5 times the volumes of 0.1 M ammonium acetate/methanol to precipitate overnight, then wash the protein pellet with methanol and acetone sequentially. Finally, the precipitate was reconstituted with 8 M urea, and protein concentration was measured by BCA kit (Tiangen, Beijing, China).

### 2.4 Trypsin digestion

An equal amount of each sample was digested, and the volume was coordinated to be consistent with the lysate. Slowly add trichloroacetic acid with a final concentration of 20%, vortex and mix adequately, and precipitate at 4,500 g for 2 h at 4°C, centrifuge for 5 min, discard supernatant, wash the pellet with pre-chilled acetone 2-3 times. After the pellet was dried, tetraethylammonium bromide with a final concentration of 200 mM was added, then the pellet was dispersed with ultrasonication, trypsin was added at a ratio of 1:50 (protease: protein, m/m) for enzymatic hydrolysis overnight. Dithiothreitol (DTT) was added to a final concentration of 5 mM and reduced at 56°C for 30 min. Iodoacetamide (IAA) was then added to a final concentration of 11 mM and incubated for 15 min at room temperature in the dark (29).

### 2.5 Enrichment of lysine-succinylated peptides

The peptides were dissolved in the IP buffer solution (100 mM NaCl, 1 mM EDTA, 50 mM Tris-HCl, 0.5% NP-40, pH 8.0), the supernatant was transferred to pre-washed succinylated resin (antibody resin, catalog number 1066450273891599422 from PTM Bio), then it was placed on a rotary shaker at 4°C and gently shaken for incubation overnight. After the incubation, the resin was washed 4 times with IP buffer solution followed by deionized water twice. Finally, the resin-bound peptide was eluated by 0.1% trifluoroacetic acid for a total of three times, and the eluate was collected and vacuum freeze-drained. After drying, it was desalted in accordance with the C18 ZipTips instructions, and vacuum freeze-dried for LC-MS/MS analyses.

### 2.6 LC–MS/MS analysis

The peptides were dissolved in LC mobile phase A and separated using the NanoElute ultra-high performance liquid phase system with mobile phase A containing 0.1% formic acid and 2% acetonitrile; mobile phase B comprised 0.1% formic acid and 100% acetonitrile. Liquid gradient setting: 0–43 min, 6–22% B; 43–56 min, 22–30% B; 56–58 min, 30–80% B; 58–60 min, 80% B, flow rate maintained at 450 nL/min. And then the peptides were injected into a capillary ion source for ionization, and analyzed by timsTOF Pro mass spectrometry. The ion source voltage was set to 1.8 kV, and the peptide precursor and its secondary fragments were detected and analyzed using high-resolution TOF. The secondary mass spectrum scan range was set to 100–1,700. Parallel cumulative serial fragmentation (PASEF) mode was used for data acquisition. After a primary mass spectrum acquisition, the secondary spectra with precursor charge numbers in the range of 0–5 was aquired by using 10 PASEF mode, and the dynamic exclusion time of tandem mass scanning was set to 30 s to avoid repeated scanning of the precursor ions.

### 2.7 Annotation methods

Gene ontology (GO): firstly, converting identified protein ID to UniProt ID and then mapping to GO IDs by protein ID (http://www.ebi.ac.uk/GOA/). If some identified proteins were not annotated by UniProt-GOA database, the InterProScan soft (http://www.ebi.ac.uk/interpro/) would be used to the annotated protein’s GO functional descrptions based on the protein sequence alignment method. Then proteins were classified by Gene Ontology annotation based on three categories: Biological Process (BP), Cellular Component (CC) and Molecular Function (MF). Identified protein’s domain functional descriptions were annotated by InterProScan (a sequence analysis application) based on the protein sequence alignment method, and the InterPro domain database was used.

Kyoto Encyclopedia of Genes and Genomes (KEGG): Firstly, KEGG online service tool KAAS was used to annotate the protein’s KEGG database description. Then KEGG online service tool KEGG mapper was used to map the annotation result on the KEGG pathway database (https://www.genome.jp/kegg/).

Subcellular localization: WoLF PSORT, a subcellular localization prediction soft and an updated version of PSORT/PSORT II (https://wolfpsort.hgc.jp/), was used to predict subcellular localization and eukaryotic sequences. Special for protokaryon species, subcellular localization prediction soft CELLO was used.

### 2.8 Functional enrichment

Enrichment of GO, KEGG or protein domain analysis: For each category, a two-tailed Fisher’s exact test was employed to test the enrichment of the differentially expressed protein against all identified proteins with a corrected p-value < 0.05 is considered significant.

### 2.9 Enrichment-based clustering

The relevant functions (such as GO, Domain, KEGG Pathway) in different groups were clustered together using hierarchical clustering based on differentially expressed protein functional classification), all the categories obtained after enrichment along with their *P* values were collated firstly, and then the categories which were at least enriched in one of the clusters with *P* value of <0.05 were filtered. This filtered *P* value matrix was transformed by the function x = −log10 (*P* value). Finally, these x values were z-transformed for each functional category. These z scores were then clustered by one-way hierarchical clustering (Euclidean distance, average linkage clustering) in Genesis. Cluster membership was visualized through a heat map using the “heatmap.2” function from the “gplots” R-package.

### 2.10 Western blotting

Protein samples were denatured at 100°C for 3 min, then were run on 12% sodium dodecyl sulfate-polyacrylamide gel electrophoresis (SDS–PAGE), and transferred to polyvinylidene fluoride membranes. After the membranes were blocked with blocking buffer for 1 h at room temperature, the membranes were probed with the pan anti-acetyllysine antibody, pan anti-succinyllysine antibody, and pan anti-crotonyllysine antibody (PTM Biolabs, Hangzhou, China) at 4°C overnight for detection of the protein expression. The membranes were washed with TBST buffer, incubated with the anti-mouse-HRP antibody for 1 h at room temperature, and imaged via ECL detection.

### 2.11 Checkerboard microdilution test

The CLSI M38-A2 broth microdilution method was used to determine the minimum inhibitory concentrations (MICs) of ITR (0.00375–0.5 μg/mL) and NAM (0.2–25.6 mg/mL) against the *A. fumigatus* isolates. The interaction between NAM and ITR was further analyzed from the fractional inhibitory concentration index (FICI), which was calculated with the equation (MIC_NAM_ in combination/MIC_NAM_ alone) + (MIC_ITR_ in combination/MIC_ITR_ alone). The interaction was classified as synergism at FICI ≤0.5; indifference at 0.5 < FICI ≤ 4; and antagonism at FICI > 4 (30). The experiment was independently repeated thrice.

### 2.12 *In vivo* experiments

Female BALB/c mice (18–22 g; Zhejiang Vital River Laboratory Animal Technology Co., Ltd., China) were used in the experiment, with free access provided to food and water. The mice were randomly assigned into groups of 10 each. For AF293, the mice were immunosuppressed with a single-dose cyclophosphamide 200 mg/kg intravenous on day 3 before infection so as to yield temporary neutropenia and then infected on day 0 via the lateral tail vein with conidial suspensions containing 1.2 × 10^6^ CFU/mouse (31). For the ITR-resistant strain group, the mice were immunosuppressed with multiple doses of cyclophosphamide 200 mg/kg intravenous every 3 d, starting on day 3 to yield persistent neutropenia, and then infected on day 0 via the lateral tail vein with a conidial suspension of 4 × 10^5^ CFU/mouse (calculated from the 90% lethal dose).

Treatment was initiated 2 h after the infection. Briefly, the mice were treated with NAM (500 mg/kg intraperitoneally daily) and/or ITR (25 mg/kg orally twice a day) for 10 d. The control mice were infected and treated with relevant vehicles. The mice wereobserved at least 4 times daily and were humanely terminated when the mobility was reduced severely or in substantial distress. The endpoint time of the survival experiment was 14 d post infection. Subsequently, all surviving mice were terminated humanely.

### 2.13 Fungal killing assay

To further understand the function of NAM in *A. fumigatus* infection, we investigated its effect on macrophage killing. Briefly, THP-1 cells were cultured in RPMI 1640 medium supplemented with 10% fetal bovine serum, 0.05 mM β-mercaptoethanol, 1% penicillin (10,000 U/mL) and streptomycin (10,000 mg/mL) at 37°C and 5% CO_2_ in a humidified incubator. PMA (100 ng/mL) was used to induce cell differentiation. THP-1 macrophages were incubated with *A. fumigatus* conidia (MOI 0.1 or 1) for 1 h at 37°C under a 5% CO_2_ atmosphere. Non-phagocytosed conidia were removed by gently washing with PBS thrice, followed by the addition of fresh medium or 20 mM NAM. The cells were returned to the incubator for 4 h to perform fungal killing. Then, the medium was removed and the cells were lysed for harvesting the conidia. The conidia suspension was diluted by gradient in a sterile medium and immediately plated on PDA plates.

### 2.14 Real-time quantitative reverse transcription polymerase chain reaction assay

Total RNA was isolated from CZZJ100527, CZZJ100527, and AF293 according to the standard methods. The cDNA was synthesized and quantitative real-time reverse transcription polymerase chain reaction was conducted using the ABI Prism 7000 Sequence Detection System (Applied Biosystems, Waltham, MA, USA). The primer sequences are listed in the **Supplementary Table**. The results were presented at a relative mRNA expression level by using the ΔΔCt method.

### 2.15 Genotyping detection of Cyp51A

The Cyp51A gene sequences of ITR-resistant strains were detected, including coding and promoter regions. The Cyp51A promoter was amplified using the primer TR34. In order to determine tandem repeats and mutation, the sequence alignment of Cyp51A was performed in the Fungal Resistance Database (FunResDB https://sbi.hki-jena.de/FunResDb) (32).

### 2.16 Statistical analyses

All statistical data were analyzed using GraphPad Prism (GraphPad Software Inc., San Diego, CA, USA). Student’s *t*-test was applied when two groups were compared and one-way ANOVA combined with a Tukey’s post-test was applied for multiple comparisons. Survival curves were compared by using the log-rank test. All data in the study were presented as a mean ± SD. Data were also expressed as a percent. The differences were statistically significant at *P* < 0.05.

## 3 Results

### 3.1 Antifungal susceptibility testing

To understand the triazole resistance of *A. fumigatus*, three common azole drugs were chosen for *in vitro* drug sensitivity tests. Posaconazole MIC was 0.03–0.5 μg/mL; the overall MIC_50_ was 0.03 μg/mL and MIC_90_ was 0.12 μg/mL. Voriconazole MIC was 0.25–1 μg/mL; the overall MIC_50_ was 0.25 μg/mL and MIC_90_ was 0.5 μg/mL. ITR MIC was 0.25–16 μg/mL; the overall MIC_50_ was 0.5 μg/mL and MIC_90_ was 1 μg/mL. The results showed that all clinical strains were sensitive to posaconazole and voriconazole, and two strains (7.4%) were resistant to ITR (**Table 1**).

**Table 1:** Antifungal susceptibility testing results of *Aspergillus fumigatus*.

Fungal name	MIC (μg/mL)			Strain source
	Posaconazole	Voriconazole	Itraconazole	
AF293	0.03	0.5	0.5	ATCC
CZZJ100508	0.03	0.5	0.5	Clinical isolation strain
CZZJ100509	0.12	0.25	0.5	Clinical isolation strain
CZZJ100510	0.06	0.5	1	Clinical isolation strain
CZZJ100511	0.03	0.25	0.5	Clinical isolation strain
CZZJ100512	0.03	0.25	0.5	Clinical isolation strain
CZZJ100513	0.03	0.25	1	Clinical isolation strain
CZZJ100514	0.03	0.5	0.5	Clinical isolation strain
CZZJ100515	0.25	0.5	0.5	Clinical isolation strain
CZZJ100516	0.03	0.25	0.5	Clinical isolation strain
CZZJ100517	0.06	0.5	0.25	Clinical isolation strain
CZZJ100518	0.06	1	0.5	Clinical isolation strain
CZZJ100519	0.03	0.25	0.25	Clinical isolation strain
CZZJ100520	0.03	0.25	0.5	Clinical isolation strain
CZZJ100521	0.03	0.25	0.25	Clinical isolation strain
CZZJ100522	0.06	0.5	0.25	Clinical isolation strain
CZZJ100523	0.03	0.5	0.5	Clinical isolation strain
CZZJ100524	0.06	1	0.5	Clinical isolation strain
CZZJ100525	0.03	0.25	0.25	Clinical isolation strain
CZZJ100526	0.06	0.5	0.25	Clinical isolation strain
CZZJ100527	0.03	0.5	16	Clinical isolation strain
CZZJ100528	0.12	0.25	8	Clinical isolation strain
CZZJ100529	0.03	0.25	1	Clinical isolation strain
CZZJ100530	0.06	0.25	0.5	Clinical isolation strain
CZZJ100531	0.06	0.25	0.5	Clinical isolation strain
CZZJ100532	0.03	0.25	0.5	Clinical isolation strain
CZZJ100533	0.25	1	0.25	Clinical isolation strain
CZZJ100534	0.5	1	0.5	Clinical isolation strain

### 3.2 Genes related to triazole resistance

**Figure 1:**
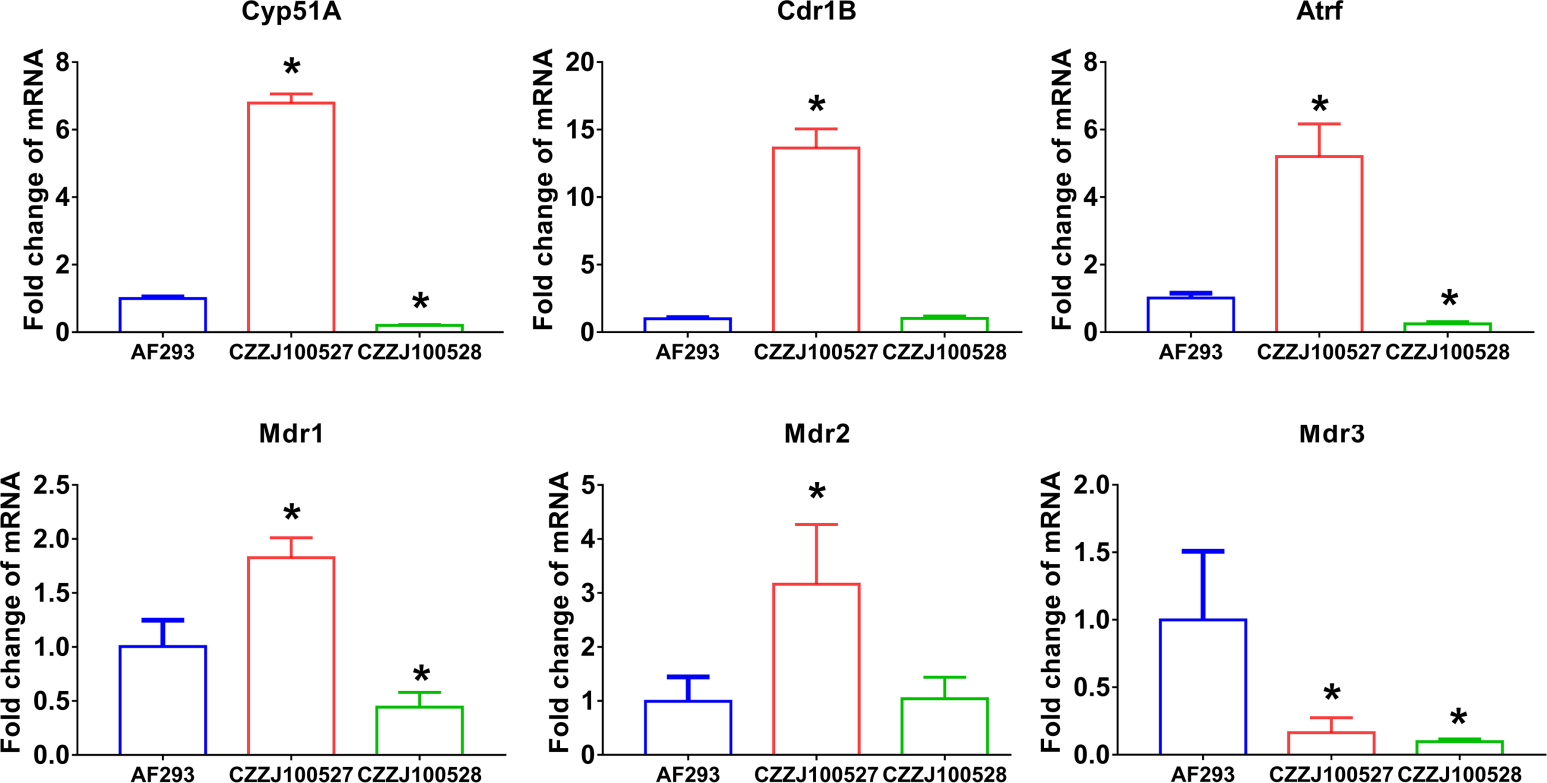
Expression of genes associated with triazole resistance mRNA levels of Cyp51A, Cdr1B, AtrF, Mdr1, Mdr2, and Mdr3 in AF293, CZZJ100527, and CZZJ100528 were determined. The genes of interest are expressed as fold change relative to that of AF293. ^*^ *P* < 0.05 vs. AF293 group.

Research on triazole resistance has mainly focused on Cyp51A mutations, Cyp51A overexpression, and efflux pump genes (33). The promoter region of CZZJ100527 was found to contain 34 short tandem repeats; moreover, the 98th amino acid in the coding region had mutated from leucine to histidine (TR34/L98H mutation) but Cyp51A of CZZJ100528 had no mutation. The mRNA levels of the drug-resistant genes Cdr1B, Mdr1, Mdr2, and AtrF were significantly higher in the drug-resistant strain CZZJ100527 than in the sensitive strain AF293, while the mRNA level of Mdr3 was significantly lower in CZZJ100527 than in AF293 (**Figure 1**). Interestingly, mRNA levels of the above mentioned genes were not elevated in the drug-resistant strain CZZJ100528 compared to sensitive strains but were instead downregulated (Cyp51A, Mdr1, Mdr3, and AtrF), suggesting that the CZZJ100528 strain may have other drug-resistant mechanisms.

### 3.3 Proteome-wide analysis of lysine succinylation sites and proteins in *A. fumigatus*

**Figure 2:**
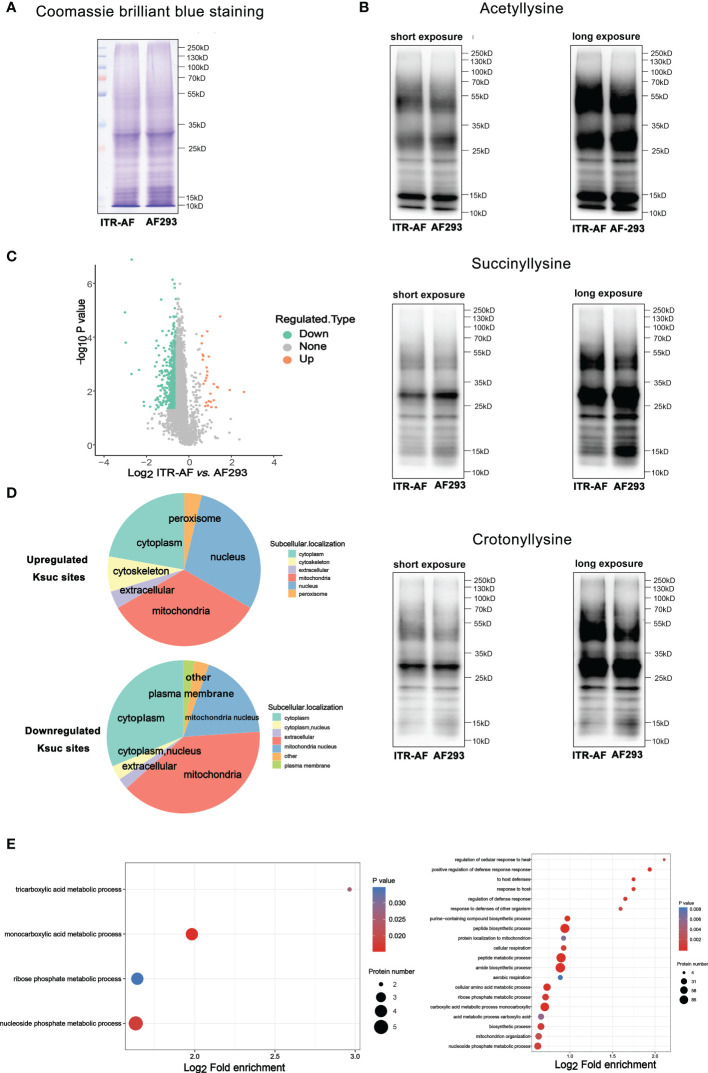
Proteome-wide analysis of lysine succinylation sites and proteins in *A. fumigatus.* **(A)** Coomassie brilliant blue staining of SDS–PAGE gel revealed the protein distribution in ITR-AF and AF293; **(B)** Western blotting revealed the acetyllysine, succinyllysine, and crotonylllysine level in ITR-AF and AF293; **(C)** Volcano plots depicting the differentially succinylated lysine sites in ITR-AF compared with that in AF293. P-value was calculated using a two-sided Wilcox rank-sum test; **(D)** Pie charts depicting the subcellular localization of differentially succinylated lysine sites in ITR-AF relative to that in AF293. P-value was calculated using a two-sided Wilcox rank-sum test; **(E)** Bubble charts describing the pathways (GO biological process) engaged with differentially succinylated lysine sites in ITR-AF when compared with that in AF293. The p-value was calculated using the hypergeometric test.

To further understand the mechanism of triazole resistance, we compared drug-resistant strain CZZJ100528 (abbreviated as ITR-AF in **Figure 2**) to sensitive strain AF293. We initially used Coomassie brilliant blue staining SDS–PAGE gel to illustrate the protein distribution in two types of *A. fumigatus* (**Figure 2A**), and then utilized western blot to evaluate several kinds of PTMs’ levels in ITR-AF and AF293 *A. fumigatus* (**Figure 2B**). The main component of succinylation modification, succinyl lysine, was discovered to alter the most dramatically. The overall succinylated proteomic data from two types of *A. fumigatus* were studied and compared. As shown in **Figure 2C**, some succinylated proteins could be considered as differentially expressed, which seemed to be responsible for the differences in drug resistance. As a result, we investigated these proteins’ subcellular localization. Both up/downregulated proteins converged in mitochondria and nucleus cytoplasm (**Figure 2D**). Interestingly, they all have one trait: rapid metabolism. The following functional analysis further confirmed the point. These proteins played roles in a series of metabolic processes. Some notable examples were tricarboxylic acid metabolic processes, monocarboxylic acid metabolic processes, and peptide metabolic processes (**Figure 2E**). The foregoing findings piqued our interest; therefore we investigated if there was any change in the cell metabolism process of drug-resistant strains.

### 3.4 Characterization of lysine succinylome sites in *Aspergillus fumigatus*

**Figure 3:**
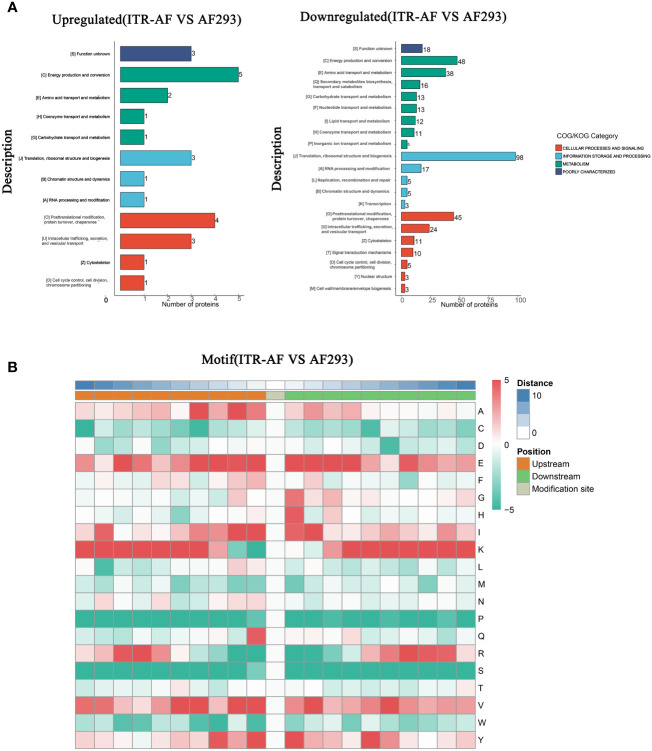
Characterization of lysine succinylome sites in *A. fumigatus.* **(A)** Barplots depicting the protein classification of differentially succinylated lysine sites in ITR-AF relative to that in AF293. The p-value was calculated using the hypergeometric test. **(B)** Heatmap illustrating the results of motif analysis.

The COG/KOG classification of these proteins revealed that the majority of both up/downregulated proteins could be located under terminology related to metabolism (**Figure 3A**). To this extent, we had sufficient reasons to speculate that succinylation modification played a necessary role in modifying metabolism and thus affecting drug resistance. In order to characterize the nature of the succinylation in *A. fumigatus*, the sequence motifs of succinylated lysines in all of the succinylated sites were analyzed using the Motif-X program, which could extract overrepresented patterns in any given set of sequences. A comparison of these motifs from succinylated peptides indicated that three residues — Glutamic acid (E), Lysine (K), and Valine (V) were commonly found around succinylated lysine (**Figure 3B**).

### 3.5 Proteomics based on LC–MS/MS showing the characterization of succinylated proteins in *Aspergillus fumigatus*

**Figure 4:**
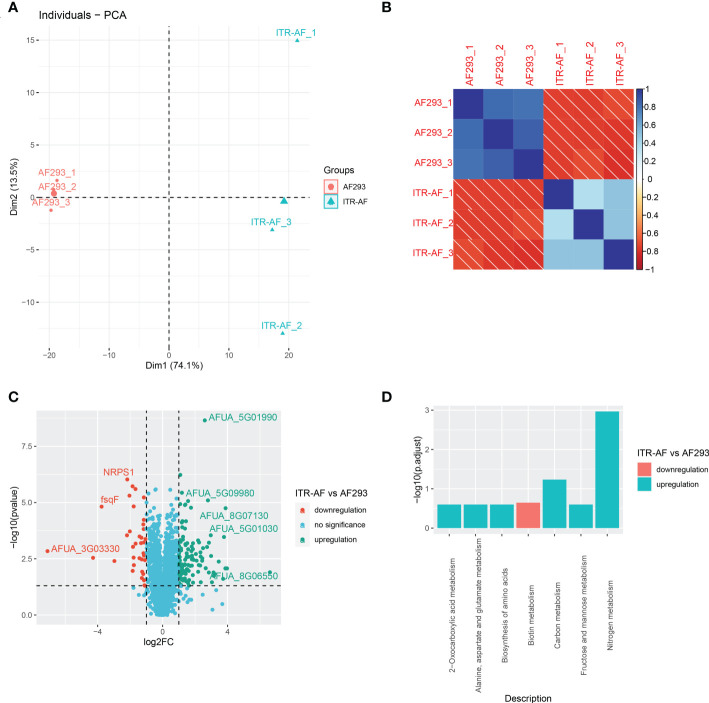
Proteomics based on LC–MS/MS showing the characterization of succinylated proteins in *A. fumigatus.* **(A)** PCA analysis of all samples based on their protein expression. **(B)** Correlation heatmap depicting the similarity of all samples. **(C)** Volcano plots depicting the differentially expressed proteins in ITR-AF relative to that in AF293. P-value was calculated with a two-sided Wilcox rank-sum test. **(D)** Bubble charts describing the pathways engaged with differential proteins in ITR-AF compared with that in AF293. The p-value was calculated by the hypergeometric test.

To further determine the protein succinylome in two kinds of *A. fumigatus* (ITR-AF and AF293), a proteomic method based on affinity purification and LC-MS/MS was then applied. In this research, 3 parallel enrichments were performed and analyzed respectively. The distribution of mass error, in general, is near zero and most of them are less than 3 ppm which satisfies the mass accuracy of the MS. Lengths of most peptides range from 8 to 20 as the typical tryptic peptides, which further confirms that the sample preparation met the requirement. The PCA analysis results in **Figure 4A** illustrated that there were proteomic differences between the two types of *A. fumigatus*. The biological repeat correlation was shown in **Figure 4B**. With the change in drug resistance, those up/downregulated proteins were detected by LC–MS/MS (**Figure 4C**). Among these, nonribosomal peptide synthetase 1 (NRPS1), a protein identified in the host’s antifungal process (34), was significantly downregulated in the drug-resistant strain. We were shocked to discover that virtually all of these differential proteins were involved in metabolic activities after doing a functional enrichment analysis on them (**Figure 4D**). Biotin metabolism, for example, was downregulated when drug resistance was enhanced, although nitrogen and carbon metabolism were upregulated.

### 3.6 NAM enhanced the sensitivity of *Aspergillus fumigatus* to ITR *in vitro*

To further understand the relationship between drug resistance and lysine succinylation, we conducted checkerboard microdilution tests to detect whether dessuccinylase inhibitor NAM (also known as a metabolic conditioner) (35) might increase *A. fumigatus*’s sensitivity of ITR. The results showed that NAM might improve not only the sensitivity of resistant strains but also the sensitivity of sensitive strains. NAM demonstrated a synergic antifungal effect against 88.8% of *A. fumigatus* clinical strains when combined with ITR. The MIC range of NAM alone was 12.8–25.6 mg/ml; however, when coupled with ITR, it was reduced to 0.2–6.4 mg/ml (**Table 2**). Meanwhile, the MIC range of ITR dropped to 0.00375–0.25 μg/ml in combination with NAM.

**Table 2:** Results of inhibition interactions between itraconazole and nicotinamide against *Aspergillus fumigatus* in the checkerboard assay.

Fungal strain	Itraconazole		Nicotinamide			
	MIC	MIC	MIC	MIC		
	(alone)	(combination)	(alone)	(combination)	FICI	IN
CZZJ100508	0.5	0.12	12.8	3.2	0.49	Synergism
CZZJ100509	0.5	0.12	25.6	6.4	0.49	Synergism
CZZJ100510	1	0.12	25.6	6.4	0.37	Synergism
CZZJ100511	0.5	0.12	25.6	1.6	0.30	Synergism
CZZJ100512	0.5	0.12	25.6	3.2	0.36	Synergism
CZZJ100513	1	0.12	25.6	0.8	0.15	Synergism
CZZJ100514	0.5	0.12	12.8	3.2	0.49	Synergism
CZZJ100515	0.5	0.12	25.6	1.6	0.30	Synergism
CZZJ100516	0.5	0.12	25.6	3.2	0.36	Synergism
CZZJ100517	0.25	0.06	25.6	6.4	0.49	Synergism
CZZJ100518	0.5	0.06	25.6	6.4	0.37	Synergism
CZZJ100519	0.25	0.12	12.8	0.2	0.49	Synergism
CZZJ100520	0.5	0.12	25.6	1.6	0.30	Synergism
CZZJ100521	0.25	0.12	25.6	0.2	0.48	Synergism
CZZJ100522	0.25	0.12	12.8	0.2	0.49	Synergism
CZZJ100523	0.5	0.12	12.8	0.2	0.25	Synergism
CZZJ100524	0.5	0.12	25.6	6.4	0.49	Synergism
CZZJ100525	0.25	0.12	25.6	0.2	0.48	Synergism
CZZJ100526	0.25	0.12	12.8	1.6	0.61	Indifference
CZZJ100527	16	0.00375	25.6	6.4	0.25	Synergism
CZZJ100528	8	0.00375	25.6	6.4	0.25	Synergism
CZZJ100529	1	0.12	25.6	0.8	0.15	Synergism
CZZJ100530	0.5	0.12	12.8	3.2	0.49	Synergism
CZZJ100531	0.5	0.12	25.6	1.6	0.30	Synergism
CZZJ100532	0.5	0.25	25.6	0.2	0.51	Indifference
CZZJ100533	0.25	0.06	25.6	6.4	0.49	Synergism
CZZJ100534	0.5	0.25	25.6	0.2	0.51	Indifference

MIC, minimal inhibitory concentration; FICI, fractional inhibitory concentration index; IN, interpretation

### 3.7 Treatment with ITR in combination NAM prolonged the survival of mice infected with *Aspergillus fumigatus*

**Figure 5:**
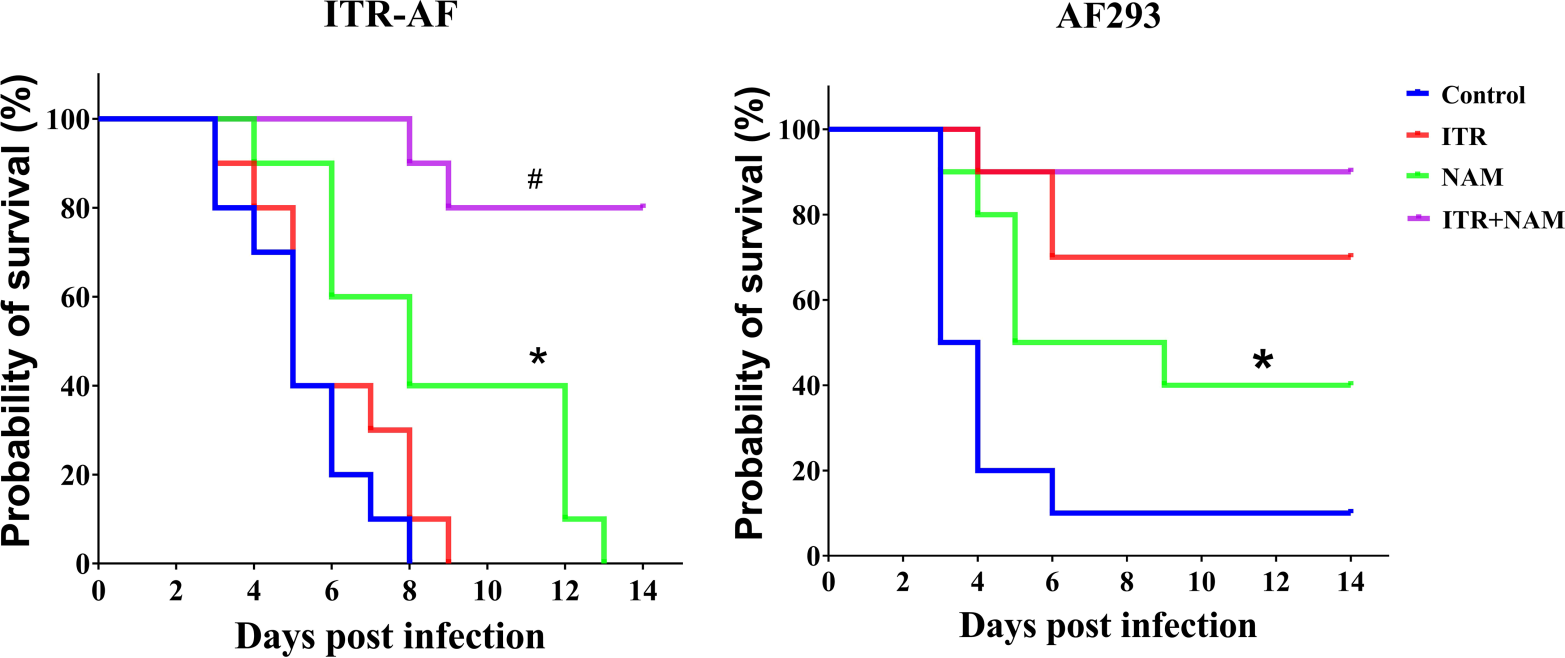
Survival curves of mice infected with the triazole-sensitive strain AF293 and triazole-resistant strain ITR-AF. Mice infected with *A. fumigatus* were treated with ITR (25 mg/kg orally twice a day) alone or in combination with NAM (500 mg/kg intraperitoneally daily). ITR, itraconazole; NAM, nicotinamide. ^*^ *P* < 0.05 vs. control group; ^#^ *P* < 0.05 vs. ITR group.

The survival curves (**Figure 5**) indicated that both the resistant strain ITR-AF and the sensitive strain AF293 caused acute and fatal infections in mice. In untreated mice, mortality was substantial after ITR-AF infection, with death occurring 5–8 d post infection. Treatment with ITR alone could not improve survival (*P >* 0.05). Survival following NAM treatment was superior to treatment with controls (*P* < 0.05). Treatment with ITR in combination with NAM resulted in 80% survival, which was significantly prolonged than ITR alone (*P* < 0.05). For the sensitive strain AF293, similar results were obtained in the treatment of NAM, which significantly prolonged survival compared with the control group (*P* < 0.05).

### 3.8 NAM enhanced the killing effect of macrophages on *Aspergillus fumigatus* conidia

**Figure 6:**
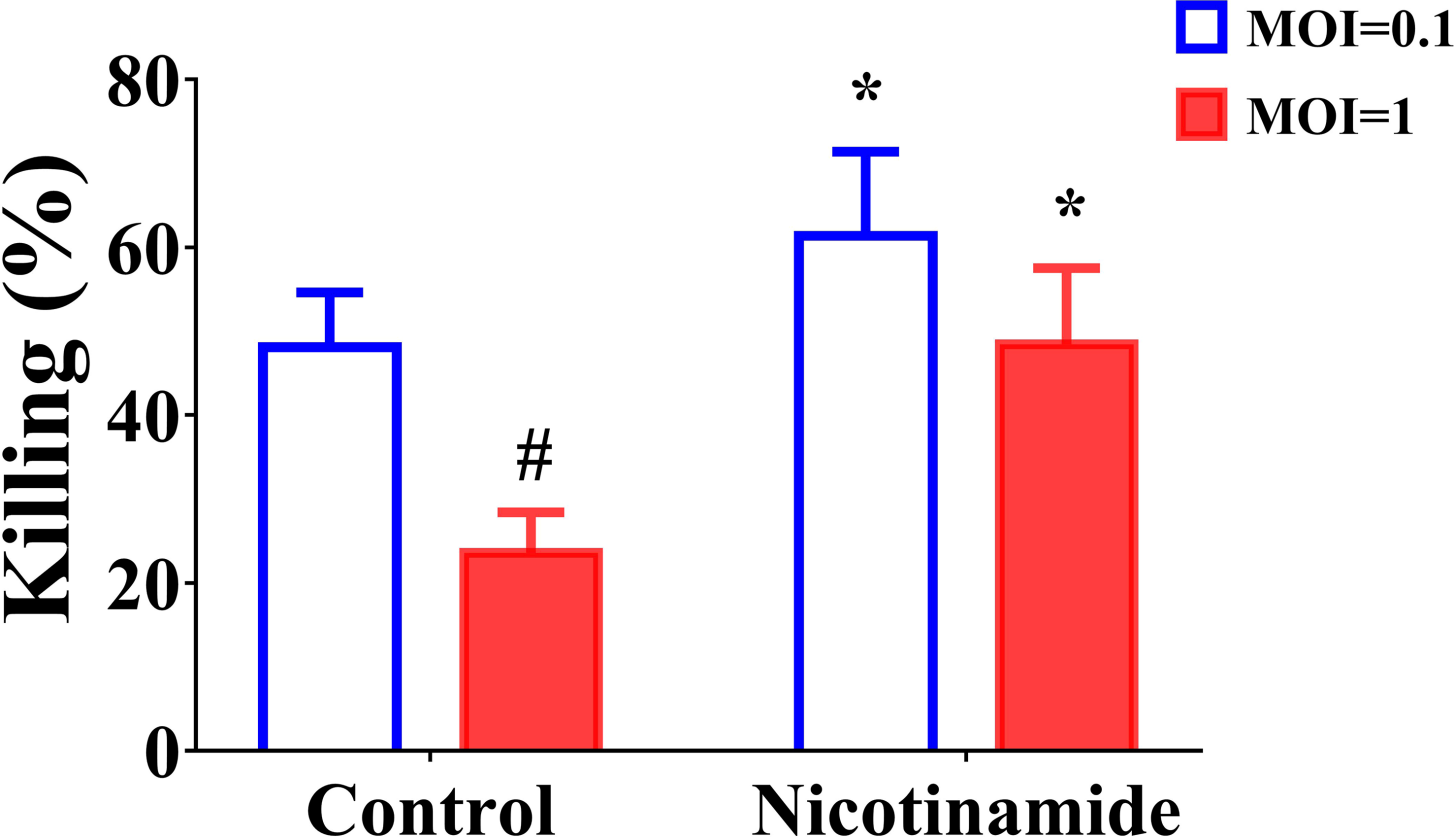
Fungal killing assay. THP-1 macrophages were incubated with MOI = 0.1 or MOI = 1 *A. fumigatus* conidia for 1 h, and a fungal killing assay was performed 4 h after the removal of the unbound conidia. ^#^ *P* < 0.05 vs. MOI = 0.1 control group; ^*^ *P* < 0.05 vs. the respective control group.

The above *in vivo* experimental results indicated that NAM alone can also exert a certain therapeutic effect. Therefore, we speculate whether NAM has other antifungal mechanisms besides synergistic antifungal effects. Macrophages play an important role in antifungal infection, such as phagocytosing and killing fungal spores (36). Therefore, we investigated the impact of NAM using an *in vitro* macrophage model. The results indicated that conidia were phagocytosed and killed by THP-1 macrophage (**Figure 6**). The NAM group killed considerably more *A. fumigatus* conidia than the control group (*P* < 0.05). We also compared killing at different MOIs. Although MOI=0.1 group killed significantly more conidia (*P* < 0.05). When NAM was introduced, killing increased in both the MOI=0.1 and MOI=1 groups (*P* < 0.05). This result shows that NAM could enhance macrophage killing of *A. fumigatus conidia.*

## 4 Discussion

Triazole resistance in *A. fumigatus* is becoming prevalent on a global scale, increasing the risk of treatment failure and mortality (37). Triazole resistance can emerge when genetic diversity occurs in the offspring of Aspergillus species and any triazole-containing environment (11). Triazole-treated individuals and surroundings with triazole fungicide residues are commonly recognized as two variables that enhance resistance selection in *A. fumigatus* (10, 38, 39). Patients with pulmonary cavities may experience resistance selection, which is caused by triazole medication.

The Cyp51A gene, which is the target of antifungal triazoles, frequently exhibits resistance mutations, which are defined by single nucleotide changes, such as G54, M220, and G138 (40, 41). Patients with chronic lung diseases, such as chronic obstructive pulmonary disease, receiving long-term triazole medication frequently develop single nucleotide resistance mutations. As a result, *A. fumigatus* with medication resistance is not merely a bystander but rather a major contributor to lung damage. Recent studies have found that an increasing amount of triazole resistance is independent of Cyp51A mutation. No mutations in the Cyp51A gene were found in 21.79% of phenotypically resistant isolates (12). Overexpression of Cyp51A and efflux pump genes were also linked to triazole resistance (42). Only one strain in our study had the TR34/L98H mutation in the Cyp51A gene. Moreover, it was not associated with the overexpression of efflux pump genes, implying the presence of additional triazole-resistant mechanisms (43).

In this study, we provided the first in-depth analysis of the lysine succinylome for the human fungal pathogen *A. fumigatus*, using a series of highly sensitive proteomic methods. AF293 is a standard strain, while ITR-AF is a drug-resistant strain, some succinylated proteins were differentially expressed, which may contribute to the difference in drug resistance. In addition, according to extensive characterization of the succinylome, we identified that the succinylated proteins were mostly associated with cellular functions and were dispersed in the different cellular organelles, which played a key role in metabolism. Succinylation also targets major metabolic pathways, including the TCA cycle, monocarboxylic acid metabolic process, and peptide metabolic process. All of these pathways are crucial components of *A. fumigatus* energy metabolic networks. We had enough evidence at this point to assume that succinylation alteration was essential for altering metabolism and influencing drug resistance.

Furthermore, by analyzing the amino acid sequence motifs, we discovered that the succinylated lysine shared three distinct types of residues: Glutamic acid (E), Lysine (K), and Valine (V). Furthermore, as drug resistance altered, those up and downregulated proteins were found. Among these, ITR-AF significantly downregulated NRPS1, a protein known to function during the host’s antifungal response (34). Using functional enrichment analysis on these proteins, the differential proteins were shown to be engaged in metabolic processes. For instance, when drug resistance developed, biotin metabolism was downregulated but nitrogen and carbon metabolism was upregulated.

NAM, a safe vitamin, is clinically used to treat pellagra, which is caused by nicotinic acid deficiency (44). Its significance in antimicrobial infections has just recently become apparent. NAM was found to inhibit the growth of *Plasmodium falciparum* (45), trypanosomes (46), *Mycobacterium tuberculosis*, human immunodeficiency virus (47), and *Candida albicans* (48). NAM in combination with fluconazole demonstrated a stronger antifungal activity by affecting fungal cell wall organization (48). Moreover, NAM could enhance amphotericin B’s antifungal activities against *C. albicans*, including another Candida spp. and *Cryptococcus neoformans*. On the one hand, it may enhance the activity of amphotericin B against biofilm; on the other hand, oxidative damage induced by amphotericin B can be strengthened by the addition of NAM (49).

In our study, NAM exerted similar antifungal functions. *In vitro* antifungal sensitivity test indicated that NAM combined with ITR exerted synergistic effects against ITR-resistant and ITR-sensitive *A. fumigatus*. This effect was associated with the role of dessuccinylase inhibition. In an *in vivo* experiment, NAM treatment had a curative effect in neutropenic mice infected with *A. fumigatus*; this might be related to NAM’s role in enhancing the killing effect of THP-1 macrophages on *A. fumigatus* conidia.

## 5 Conclusions

The succinylation of drug-resistant strains of *A. fumigatus* can aid in better understanding the mechanism of drug resistance and in the identification of therapeutic targets. Patients with *A. fumigatus* infection will benefit the most from this information. To the best of our knowledge, this is the first succinylome identified in the human fungal pathogen *A. fumigatus* which may serve as an important starting point for further characterization of the pathophysiological role of lysine succinylation in *A. fumigatus*. This study addressed a gap in our understanding of the triazole-resistant mechanism of *A. fumigatus*. Furthermore, the function of dessuccinylase inhibitor NAM in antifungal treatment may be studied in the future.

## Data availability statement

The datasets presented in this study can be found in online repositories. The names of the repository/repositories and accession number(s) can be found below: PXD040137 (iProX).

## Ethics statement

The animal study was reviewed and approved by Committee on Ethics of Medicine, Naval Medical University, PLA.

## Author contributions

All authors contributed to the study conception, design and data collection. XX, HC, and MD concepted and designed the study. XC, WL, and HM wrote the first draft of the manuscript. YJ and SZ were responsible for data collection and statistics. XX and MD revised the paper. All authors contributed to the article and approved the submitted version.
